# Machine learning‐based identification of cuproptosis‐related markers and immune infiltration in severe community‐acquired pneumonia

**DOI:** 10.1111/crj.13633

**Published:** 2023-06-06

**Authors:** Shuyang Chen, Zheng Zhou, Yajun Wang, Shujing Chen, Jinjun Jiang

**Affiliations:** ^1^ Department of Pulmonary and Critical Care Medicine, Zhongshan Hospital Fudan University Shanghai China; ^2^ Shanghai Respiratory Research Institute, Zhongshan Hospital, Fudan University Shanghai China

**Keywords:** ARDS, bioinformatics, cuproptosis, severe CAP

## Abstract

**Background:**

Severe community‐acquired pneumonia (SCAP) is one of the world's most common diseases and a major etiology of acute respiratory distress syndrome (ARDS). Cuproptosis is a novel form of regulated cell death that can occur in various diseases.

**Methods:**

Our study explored the degree of immune cell infiltration during the onset of severe CAP and identified potential biomarkers related to cuproptosis. Gene expression matrix was obtained from GEO database indexed GSE196399. Three machine learning algorithms were applied: The least absolute shrinkage and selection operator (LASSO), the random forest, and the support vector machine‐recursive feature elimination (SVM‐RFE). Immune cell infiltration was quantified by single‐sample gene set enrichment analysis (ssGSEA) scoring. Nomogram was constructed to verify the applicability of using cuproptosis‐related genes to predict the onset of severe CAP and its deterioration toward ARDS.

**Results:**

Nine cuproptosis‐related genes were differentially expressed between the severe CAP group and the control group: ATP7B, DBT, DLAT, DLD, FDX1, GCSH, LIAS, LIPT1, and SLC31A1. All 13 cuproptosis‐related genes were involved in immune cell infiltration. A three‐gene diagnostic model was constructed to predict the onset of severe CAP: GCSH, DLD, and LIPT1.

**Conclusion:**

Our study confirmed the involvement of the newly discovered cuproptosis‐related genes in the progression of SCAP.

## Graphical abstract

Cuproptosis is a novel form of copper‐induced mitochondrial cell death. Researchers for the first time used three machine learning algorithms to identify cuproptosis‐related genes (CRGs) in severe community‐acquired pneumonia (SCAP), a major cause of acute respiratory distress syndrome (ARDS). This article found that CRGs were involved in immune cell infiltration and constructed a three‐gene model to predict SCAP onset.

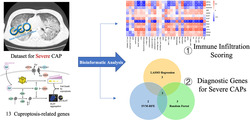

## 1 INTRODUCTION

Community‐acquired pneumonia (CAP) is a leading cause of substantial morbidity and mortality across the world. The Global Burden of Disease (GBD) study group concluded that lower respiratory tract infection is responsible for 4.4% of all deaths in people of all ages in 2016.^1^ If undertreated or severely deteriorated, CAP can easily lead to acute respiratory distress syndrome (ARDS), causing even greater damage to patients.^2^

The severity of CAP ranges from mild discomfort to severe necrotizing pneumonia with septic shock or even requiring mechanical ventilation. Studies around the globe have demonstrated the impacts of SCAP. SCAP was related to nearly 15% of in‐hospital mortality in Africa.^3^ In the United States, among 7449 patients enrolled during 2014 to 2016, 30‐day mortality of SCAP was 6%.^4^

Several scoring systems have been developed to predict the risks for severe community‐acquired pneumonia (SCAP), including Infectious Disease Society Association/American Thoracic Society (IDSA/ATS) severity score, CURB‐65, and pneumonia severity index (PSI).^5^ Most systems take key pathophysiological indexes into account, such as respiratory rate, arterial oxygen tension/fractional inspired oxygen ratio (PaO_2_/FiO_2_), yet no effective biomarker has been proposed to predict the risks for SCAP.^6^

Copper (Cu) is an essential trace element that plays a key role in many biological processes; its inherent oxidation–reduction property makes it both beneficial and potentially harmful to cells. Intracellular copper concentrations are kept at low levels through dedicated homeostatic mechanisms to avoid copper toxicity.^7^ Recently, a novel form of regulated cell death, unlike apoptosis or ferroptosis, is reported as cuproptosis. It is established that excess intracellular copper can lead to the aggregation of lipoylated proteins and destabilization of Fe‐S cluster proteins, eventually resulting in proteotoxic stress and cell death.^8^

Immune resistance is involved throughout the development of SCAP with the aim to eradicate invading pathogens. Anatomical barriers such as mucociliary clearance form the first line of defense against the pathogens.^9^ As infections progress and reach the lower respiratory tract, various immune cells, both resident and recruited, are activated to defend against the pathogens. Invading pathogens and host‐derived molecules (e.g., cytokines) can trigger lung epithelial cells through a variety of receptors and thus become a major driver both of protective immunity as well as postinjury repair.^10^ Although innate and adaptive immunities are both of great significance in diminishing the invading pathogens, immune resistance can easily inflict damage and even cause airway remodeling.^11^ It has been argued that the overactivation of the immune system is a contributing factor to the development of ARDS.^12^ Consequently, further study into the participation of immune system during SCAP is much needed.

With the help of bioinformatic analysis and novel sequencing technology, potential biomarkers can be identified and can shed light on new therapeutic possibilities. However, the study into the correlation between SCAP and cuproptosis‐related genes (CRGs) is still lacking. Our study applied the ssSGEA method to examine the immune infiltration in SCAP and investigated the differentially expressed genes (DEGs) in SCAP patients, proposing a group of genes with potential diagnostic significance for SCAP. We believe our findings can benefit the understanding of the characterization of cuproptosis progression in SCAP and allow for the development of future therapeutic approaches from this standpoint.

## 2 METHODS AND MATERIALS

### 2.1 Data acquisition

“Severe CAP” was used as a keyword to retrieve records on the NCBI Gene Expression Omnibus (GEO; https://www.ncbi.nlm.nih.gov/geo/). Inclusion criteria were set to further screen the datasets: (1) Human‐originated samples, (2) expression profiling arrays by high throughput sequencing, and (3) sample size greater than 70. One SCAP dataset indexed as GSE196399 was then downloaded. GSE196399 is a gene expression array, generated using platform GPL24676 (Illumina NovaSeq 6000). GSE196399 comprises blood samples from 21 healthy controls and 56 SCAP cases. Diagnosis of SCAP was made based on IDSA/ATS 2007 Guidelines.^13^

### 2.2 Data preprocessing and DEGs identification

As the original data were demonstrated using ensemble ID, we transformed it into gene symbol for optimal screening. R package “edgeR”^14^ was used to perform background calibration and log2^15^ transformation. All probes were unique, and no replicated correspondence was found. Cuproptosis‐related genes (CRGs) were extracted from a previous study.^8^ R package “limma”^16^ was then applied to identify DEGs. In reference to previous studies, cut‐off points for DEGs were set as adjusted *p* values <0.05 and |log2 Fold change (FC)| > 0.2.^17^

### 2.3 Immune infiltration analysis

Single‐sample gene set enrichment analysis (ssGSEA) was performed to assess the infiltrating scores of 13 immune‐related pathways and 16 types of immune cells^18^ using R package “gsva.” Subsequently, the correlation between CRGs and both immune cells and function in GSE196399 samples was examined and assessed. R package “corrplo” was used to perform correlation analysis between different immune cells and immune function. “ggpubr” package was applied to evaluate the differences of immune infiltration between SCAP patients and healthy control using the Wilcoxon rank sum test.

### 2.4 Identification of characteristic CRGs

LASSO regression analysis and random forest were applied in the screening of characteristic CRGs. As a dimensionality reduction method, LASSO regression analysis was performed using R package “glmnet”^19^ with a penalty parameter set as 10‐fold cross‐verification. The random forest algorithm was able to perform recursive feature elimination and consequently rank the CRGs in SCAP. Genes with relative importance greater than 0.25 were identified. Intersecting results from previous methods were then fit into logistic regression to finally identify characteristic CRGs.

### 2.5 Statistical analysis

All analyses were performed using R Studio version 4.2.1 and its supporting packages. The differences in immune pathways and immune cells were evaluated by the Wilcoxon rank sum test. R packages including “pheatmap,” “ggpubr,” and “ggboxplot” were used for visualization. R package “rms” was used to create a nomogram for the diagnosis of SCAP. In all statistical analyses, *p* < 0.05 was considered of statistical significance.

## 3 RESULTS

### 3.1 Identification of DEGs

**FIGURE 1:**
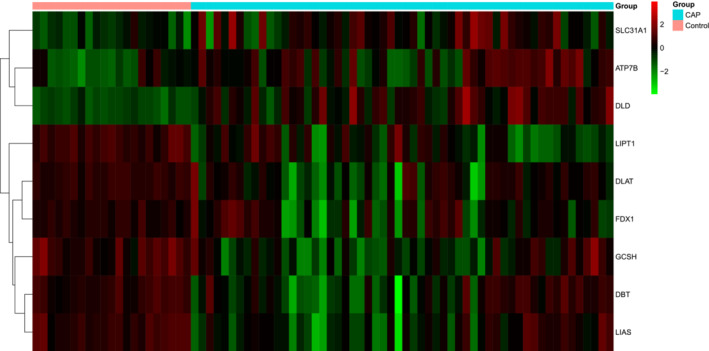
Heat map of differentially expressed cuproptosis‐related genes in GSE196399. The red color indicates an upregulation, whereas the green color indicates an downregulation.

R package “limma” was used to analyze the differentially expressed CRGs in GSE196399. Nine CRGs were differentially expressed: ATP7B, DBT, DLAT, DLD, FDX1, GCSH, LIAS, LIPT1, and SLC31A1. ATP7B, DLD, and SLC31A1 were upregulated in SCAP samples, whereas GCSH, LIPT1, LIAS, DBT, DLAT, and FDX1 were downregulated (Figure 1).

### 3.2 Immune infiltration scoring

**FIGURE 2:**
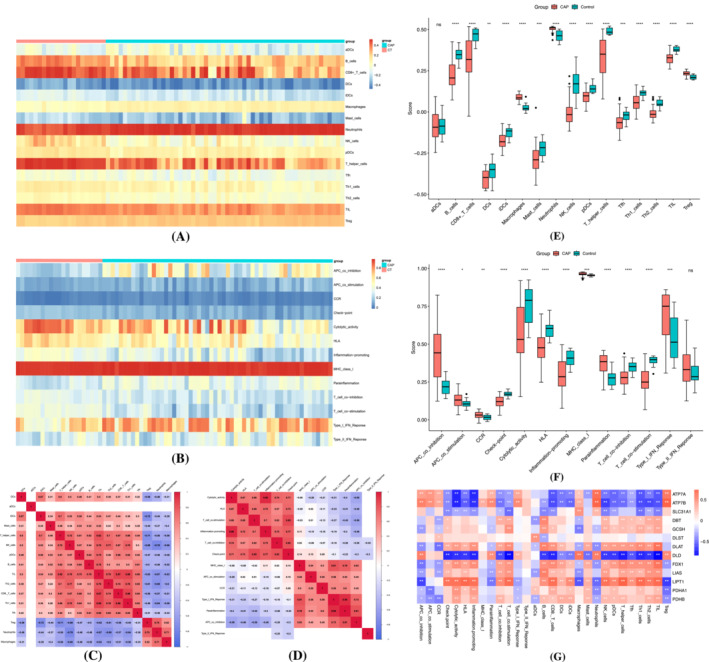
Immune infiltration analysis. (A) Heat map of immune pathways in severe community‐acquired pneumonia (SCAP) group and control group. (B) Heat map of immune cells in SCAP group and control group. (C) Correlation matrix of immune cells. (D) Correlation matrix of immune pathways. (E) Comparison of the degree of immune cell infiltration in SCAP group and control group. (F) Comparison of the degree of immune pathways involvement in SCAP group and control group. (G) Correlation analysis of cuproptosis‐related genes and immune cells and pathways. *p < 0.05, **p < 0.01, ***p < 0.001, ns no significance.

To better assess the immune microenvironment of SCAP, R package “gsva” was applied to quantify ssGSEA enrichment scores for 13 immune cell subpopulations and 16 immune‐related pathways (Figure 2A,B).

We subsequently analyze the correlation between immune cells and immune pathways (Figure 2C,D). For all immune cell subpopulations, a strong positive correlation can be observed between Th1 cells and tumor‐infiltrating lymphocytes (TIL) (*r* = 0.88), CD8^+^ T cells and Th1 cells (*r* = 0.88), and CD8^+^ T cells and TIL (*r* = 0.87). A strong negative correlation can be observed between neutrophils and NK cells (*r* = −0.76) and immature dendric cells (iDC) and regulatory T cells (Treg) (*r* = −0.72). For all immune‐related pathways, a strong positive correlation can be observed between inflammation promoting and cytolytic activity (*r* = 0.96) and parainflammation and Type 1 IFN response (*r* = 0.95). A strong negative correlation can be observed between APC co‐inhibition and cytolytic activity (*r* = −0.56), APC co‐inhibition and T cell co‐stimulation (*r* = −0.56).

We further compared the differences in ssGSEA scores between SCAP group and control group (Figure 2E,F) and found that macrophages, neutrophils, and Treg are more abundant in the SCAP group, whereas B cells, CD8^+^ T cells, DCs, iDCs, mast cells, NK cells, pDC cells, T helper cells, Tfh, Th1 cells, Th2 cells, and TIL are more abundant in the normal group. For immune‐related pathways, it was observed that APC co‐inhibition, APC co‐stimulation, chemokine receptors (CCR), MHC class I, parainflammation, and Type I and Type II IFN response were enriched in the SCAP group. Check point, cytolytic activity, HLA, inflammation promoting, T cell co‐inhibition, and T cell co‐stimulation were higher in the normal group.

We lastly performed a correlation analysis between 13 previously reported CRGs and immune infiltration (Figure 2G). It can be observed that all 13 CRGs participate and act in the microenvironment of SCAP.

### 3.3 Identification of characteristic genes

**FIGURE 3:**
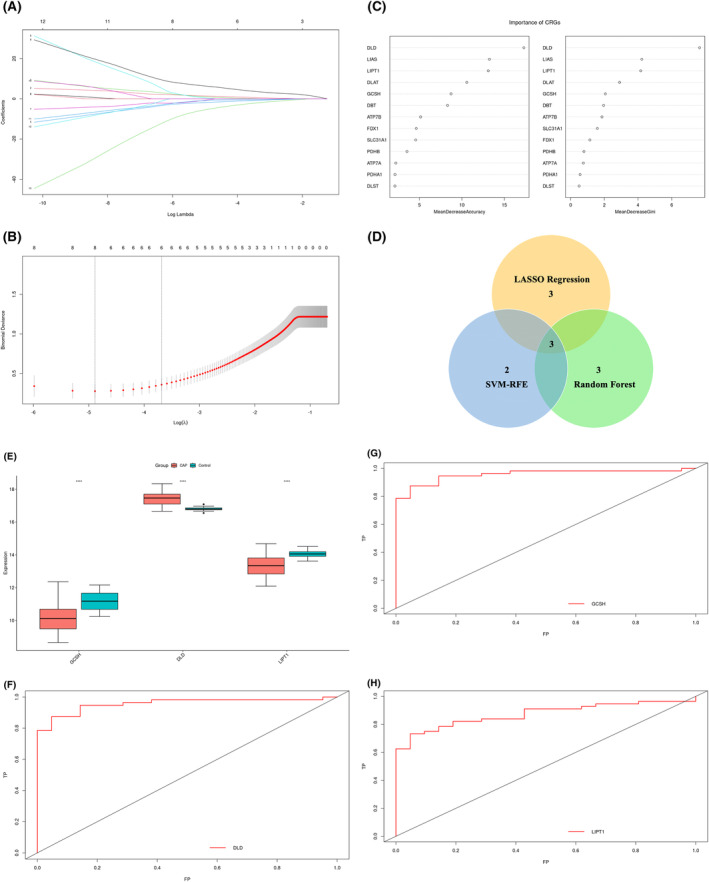
Machine learning‐based selection and identification of cuproptosis‐related genes (CRGs) associated with severe community‐acquired pneumonia (SCAP). (A) Ten cross‐validations of adjusted parameter selection in the least absolute shrinkage and selection operator (LASSO) model. (B) LASSO coefficient analysis. (C) Ranking of the relative importance of genes. (D) Venn diagram indicating the characteristic genes selected by LASSO, random forest and SVM‐RFE algorithms. (E) Comparison of signature gene expression between SCAP group and control group. (F, G, H) ROC curves for estimating the diagnostic performance of the signature genes DLD, GCSH, and LIPT1. *p < 0.05, **p < 0.01, ***p < 0.001, ns no significance.

In order to select CRGs to signify the onset of SCAP, methods including LASSO‐COX regression operation, random forest, and logistic regression were applied. A total of six genes including ATP7B, SLC31A1, GCSH, DLD, LIAS, and LIPT1 were identified by the LASSO algorithm as of diagnostic significance (Figure 3A,B). The random forest algorithm was able to identify five genes with relative importance greater than 2, including DLD, LIAS, LIPT1, DLAT, and GCSH (Figure 3C). For the SVM‐RFE algorithm, a minimal error rate was reached when the number of features was five, namely, GCSH, DLD, LIPT1, DLAT, and DLST. The intersection of three algorithms produced three characteristic genes, GCSH, DLD, and LIPT1 (Figure 3D).

We then made an attempt to verify the diagnostic efficacy of the above three genes. All three genes expressed differently between the SCAP group and control group (Figure 3E, *p* < 0.05). Among them, GCSH and LIPT1 were downregulated in the SCAP group, whereas DLD was upregulated in the SCAP group. Receiver operating characteristic (ROC) curves for three genes were composed to verify the diagnostic significance for SCAP. AUC values for ROC curves were 0.9566 for DLD (Figure 3F), 0.8359 for GCSH (Figure 3G), and 0.8724 for LIPT1 (Figure 3H).

### 3.4 Nomogram for prediction of SCAP

**FIGURE 4:**
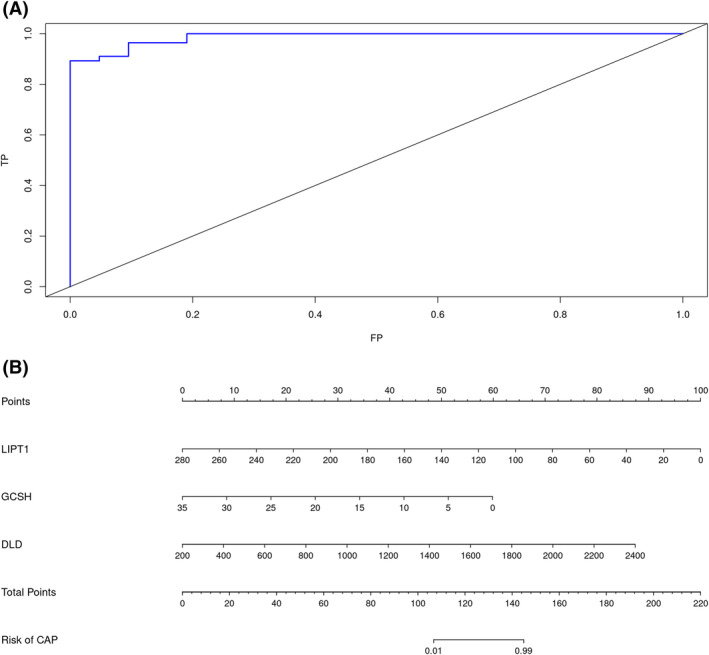
Diagnostic efficacy of signature cuproptosis‐related genes. (A) Receiver operating characteristic (ROC) curve indicating the diagnostic efficacy of signature genes. (B) Construction of column line graph integrating signature cuproptosis‐related genes (CRGs) for severe community‐acquired pneumonia (SCAP).

We subsequently composed three genes into one variable to verify its diagnostic possibility. The AUC for the composed ROC curve was 0.9872 (Figure 4A), suggesting satisfying diagnostic efficacy in predicting the onset of SCAP. Combining both clinical characteristics and gene expressions, a nomogram was constructed (Figure 4B). In this graph, the expression value of each gene can correspond to a score; a total score can be obtained by summing the scores of all three genes. This total score corresponds to different risks of SCAP.

## 4 DISCUSSION

SCAP is one of the most common diseases and a leading cause of mortality across the world. If not properly treated, SCAP can easily lead to acute respiratory distress syndrome (ARDS). ARDS is a highly heterogeneous disease, and different etiology of ARDS can produce vastly different clinical presentations and outcomes. There have been many conjectures toward the pathophysiological processes of ARDS, but no previous studies have examined the correlation between cuproptosis and the development of SCAP and ARDS. Out of 13 CRGs, nine were differentially expressed between SCAP cases and the control, suggesting a potential role of cuproptosis in the onset of SCAP and ARDS.

The roles metal ions play in the development and onset of diseases have always interest researchers. Copper, for example, participates in multiple physiological processes of great necessity to human life.^20^ The copper imbalance has been mostly attributed to the pathogenesis of neurodegenerative disorders and metabolic diseases, whereas its involvement in pneumonia and ARDS has not been fully elucidated. Previous studies mainly focus on the antimicrobial ability of copper, both directly and indirectly. It has been found that copper can act as an active effector against urinary tract infection pathogens, abrogating the propagation of invasive pathogens.^21^ Because of the essentiality of copper ions for organisms, the human body can therefore withhold the provision of copper to invasive pathogens and achieve a process termed nutritional immunity.^22^ In studies of bacterial infections, an increase in intracellular concentration of copper in certain subcellular compartments such as phagolysosome of phagocytic cells can be observed,^23^ which in turn boosts the antimicrobial responses against invasive pathogens.

Notwithstanding multiple lines of evidence suggesting the antimicrobial competence of copper, the discovery of cuproptosis allows for studying the impacts of copper from a new intracellular standpoint. Most current researches into cuproptosis focus on its impact on oncological diseases such as hepatocellular carcinoma^24^ and pancreatic cancer.^25^ Previous studies have confirmed the participation of other programmed cell death pathways in various diseases. For example, inhibition of ferroptosis can alleviate sepsis‐induced acute lung injury.^26^ Also, inflammatory caspase activation can trigger pyroptosis and the attenuation of pyroptosis can protect against LPS‐induced acute lung injury.^27^ However, cuproptosis's role in the pathogenesis of ARDS remains unclear. Because of the critical role cell death plays in the development of ARDS, it is of vital necessity to not only further investigate the pathophysiological role of cuproptosis in ARDS but also consider the highly interconnected pathways of ferroptosis, pyroptosis, and necroptosis.

Inflammatory response and immune regulation are actively involved during the onset and pathogenesis of SCAP and ARDS.^28^ Similar to methods described previously,^29^ we closely examine the correlation between the onset of diseases and the response of immune system. In our study, a strong relationship can be observed between various immune cells and the expression of CRGs. For example, infiltration of macrophages can be observed in ARDS samples. Macrophages can be further divided into M1 and M2 phenotypes, displaying proinflammatory and anti‐inflammatory ability, respectively.^30^ The balance between M1 and M2 macrophages is of great significance in the regulation of inflammatory response during ARDS. We hypothesize that cuproptosis‐determined cell death may be involved to achieve such balance.

Out of all 13 cuprotosis‐related genes, three genes (GCSH, DLD, LIPT1) were selected as signature genes in the development of SCAP based on three algorithms. All three genes were differentially expressed in SCAP and control groups.

GCSH encodes glycine cleavage system protein H, which is among the only four enzymes in which protein lipoylation can occur.^8^ Glycine cleavage system proteins catalyze the degradation of glycine. Defective glycine cleavage activity can result in nonketotic hyperglycinemia.^31^ GCSH encoding protein H can go beyond the cleavage system and has an impact on embryonic viability.^32^ However, GCSH's role in the development of pulmonary diseases has not been fully interpreted. We hypothesize that the upregulation of GCSH in the SCAP group may be due to the increased need for energy during infection and inflammation.

DLD and LIPT1 encode components of lipoic acid pathways (dihydrolipoamide dehydrogenase [DLD], lipolytransferase 1 [LIPT1]). Interestingly enough, DLD has been confirmed to be able to regulate cystine deprivation‐induced ferroptosis in head and neck cancer.^23^ LIPT1 works in the process of transferring lipoic acid to proteins. Mutations of LIPT1 have been found to be associated with a defect in the 2‐ketoacid dehydrogenase complexes.^33^ It is worth noticing that both DLD and LIPT1 are essential for cuproptosis, as they encode proteins that engage in lipoylation.

Our work is a new attempt to connect cuproptosis and respiratory diseases. By using three machine learning algorithms, we were able to construct a three‐gene predicting method that can help in the diagnosis of SCAP and its deterioration toward ARDS. However, there still exist certain limitations in our study. First of all, the applicability of our method demands future confirmation as the raw data are limited. Second, this GSE database did not provide clinical data clarifying the invasive pathogens of each patient, and there may be overlapping or differences in final results. Overall, research into cuproptosis‐related genes remains scarce. Future studies should include more patients and provide more detailed clinical data to help better understand the relationship between cuproptosis and respiratory diseases.

## 5 CONCLUSION

Our work connects SCAP and the newly identified cuproptosis for the first time. We successfully identify three cuproptosis‐related genes GCSH, DLD, and LIPT1 in the development of SCAP. The novelty of cuproptosis calls for continuous studies to further elucidate the exact pathophysiological involvement in the onset of diseases.

## AUTHOR CONTRIBUTIONS

Jinjun Jiang and Shujing Chen designed the study. Yajun Wang and Shuyang Chen searched and retrieved GEO dataset. Shuyang Chen and Zheng Zhou performed the bioinformatic analysis and analyzed the data. Shuyang Chen and Zheng Zhou drafted the manuscript. Shujing Chen and Yajun Wang revised the manuscript. Jinjun Jiang and Shujing Chen approved the submitted version. All authors contributed to the article. All authors have read and agreed to the published version of this manuscript.

## CONFLICT OF INTEREST STATEMENT

The authors declare no conflicts of interest.

## ETHICS STATEMENT

This research was conducted using publically available datasets. No potentially identifiable human images or data is presented in this study.

## Data Availability

The data that support the findings of this study are available in Gene Expression Omnibus at https://www.ncbi.nlm.nih.gov/geo/. These data were derived from the following resources available in the public domain: GSE196399, https://www.ncbi.nlm.nih.gov/geo/query/acc.cgi?acc=GSE196399.
